# Supplementary material for: Bacterial skin colonization with a specific Cutibacterium avidum clade as a risk factor for periprosthetic joint infections—a multicenter study
Source: Microbiol Spectr. 2025 Sep 26;13(11):e00515-25. doi: 10.1128/spectrum.00515-25 (PMC12584708; doi:10.1128/spectrum.00515-25)

**Supplementary Material**

**Bacterial skin colonization with a specific *Cutibacterium avidum* clade as a risk factor for periprosthetic joint infections - a multi-center study**

Keywords: *Cutibacterium*, prosthetic joint infection, biofilm, antimicrobial susceptibility

**Authors**:

Llanos Salar Vidal*^1,2^, Julia Prinz*^3^, Pascal M. Frey^4,7^, Tiziano A. Schweizer^3^, Laura Böni^4^, Silvio D. Brugger^4^, Holger Brüggemann^**5^, Jaime Esteban^**1,2^, Yvonne Achermann^**3,4,6^ on behalf of the ESGIAI (ESCMID Study Group for Implant-Associated Infections)

Contributed equally as first author (*) or last (**) author

^1^ Department of Clinical Microbiology. IIS—Fundación Jiménez Díaz, Madrid, Spain

^2^ CIBERINFEC-CIBER de Enfermedades Infecciosas, Instituto de Salud Carlos III, Madrid, Spain

^3^Department of Dermatology, University Hospital Zurich, Zurich, Switzerland

^4^Department of Infectious Diseases, University Hospital Zurich, University of Zurich, Zurich, Switzerland

^5^ Department of Biomedicine, Aarhus University, 8000 Aarhus, Denmark; brueggemann@biomed.au.dk

^6^ Hospital Zollikerberg, Zollikerberg, Switzerland

^7^ Department of General Internal Medicine, Bern University Hospital (Inselspital), University of Bern, Bern, Switzerland

* Correspondence: Yvonne Achermann, Yvonne.Achermann@usz.ch

**Supplementary Table 1**. Clinical data 11 *C. avidum* isolates from patients with a *C. avidum* PJI.

| **Countryofisolatedstrain** | **Strainlabeling** | **Joint** | **Age at infection** | **Sex** | **Immuno-suppression** |
| --- | --- | --- | --- | --- | --- |
| Slovenia | ESL2 | Hip | 60 | Female | No |
| Slovenia | ESL6 | Hip | 58 | Male | No |
| Slovenia | ESL7 | Hip | 77 | Male | No |
| Slovenia | ESL12 | Hip | 86 | Female | No |
| Netherlands | HOL4 | Knee | 23 | Male | No |
| Netherlands | HOL5 | Hip | 65 | Male | MorbusBehcet |
| Zurich | CI855_ZH09 | Hip | 64 | Female | No |
| Zurich | Y66_ZH37 | Hip | 79 | Female | No |
| Zurich | CI878_ZH40 | Hip | 59 | Female | No |
| Zurich | CI882_ZH41 | Hip | 53 | Female | No |
| Zurich | CI828_ZH42 | Hip | 81 | Male | No |

**Supplementary Table 2.** Sequence information regarding strains of *C. avidum* in this cohort (11 PJI isolates (A) 32 healthy skin isolates (B))

**A: PJI isolates**

| **Country** | **Strain** | **Contigs** | **Length (Mbp)** | **Genbank accession** |
| --- | --- | --- | --- | --- |
| Slovenia | ESL2 | 17 | 2.512 | JAHDTS |
| Slovenia | ESL6 | 20 | 2.506 | JAHDTR |
| Slovenia | ESL7 | 31 | 2.572 | JAHDTQ |
| Slovenia | ESL12 | 20 | 2.501 | JAHDTP |
| Holland | HOL4 | 22 | 2.605 | JAHDTW |
| Holland | HOL5 | 16 | 2.471 | JAHDTV |
| Switzerland | CI855_ZH09 | 23 | 2.512 | JAHDTU |
| Switzerland | Y66_ZH37 | 29 | 2.595 | JAHDTT |
| Switzerland | CI878_ZH40 | 73 | 2.541 | NBIO |
| Switzerland | CI882_ZH41 | 66 | 2.497 | NBIP |
| Switzerland | CI828_ZH42 | 30 | 2.476 | NBIQ |

**B: Skin isolates**

| **Country** | **Strain** | **Contigs** | **Length (Mbp)** | **Genbank accession** |
| --- | --- | --- | --- | --- |
| Spain | HS1 | 26 | 2.646 | JAHDUE |
| Spain | HS2 | 22 | 2.580 | JAHDUD |
| Spain | HS4 | 49 | 2.728 | JAHDUC |
| Spain | HS6 | 19 | 2.643 | JAHDUB |
| Spain | HS7 | 42 | 2.691 | JAHDUA |
| Spain | HS8 | 26 | 2.628 | JAHDTZ |
| Spain | HS9 | 37 | 2.686 | JAHDTY |
| Switzerland | PAVI-2017310049 | 27 | 2.497 | JBAKUR01 |
| Switzerland | PAVI-2017310081 | 21 | 2.501 | JBAKUQ01 |
| Switzerland | PAVI-2017310082 | 19 | 2.503 | JBAKUP01 |
| Switzerland | PAVI-2017310084 | 77 | 2.662 | JBAKUO01 |
| Switzerland | PAVI-2017310093 | 34 | 2.556 | JBAKUN01 |
| Switzerland | PAVI-2017310113 | 15 | 2.531 | JBAKUM01 |
| Switzerland | PAVI-2017310114 | 36 | 2.587 | JBAKUL01 |
| Switzerland | PAVI-2017310120 | 17 | 2.613 | JBAKUK01 |
| Switzerland | PAVI-2017310132 | 40 | 2.662 | JBAKUJ01 |
| Switzerland | PAVI-2017310145 | 55 | 2.667 | JBAKUI01 |
| Switzerland | PAVI-2017310153 | 30 | 2.657 | JBAKUH01 |
| Switzerland | PAVI-2017310154 | 27 | 2.654 | JBAKUG01 |
| Switzerland | PAVI-2017310158 | 46 | 2.605 | JBAKUF01 |
| Switzerland | PAVI-2017310163 | 28 | 2.521 | JBAKUE01 |
| Switzerland | PAVI-2017310172 | 26 | 2.545 | JBAKUD01 |
| Switzerland | PAVI-2017310183 | 32 | 2.547 | JBAKUC01 |
| Switzerland | PAVI-2017310185 | 36 | 2.548 | JBAKUB01 |
| Switzerland | PAVI-2017310195 | 55 | 2.681 | JBAKUA01 |
| Switzerland | PAVI-2017310214 | 63 | 2.567 | JBAKTZ01 |
| Switzerland | PAVI-2017310242 | 34 | 2.554 | JBEDRZ01 |
| Switzerland | PAVI-2017310243 | 23 | 2.547 | JBAKTY01 |
| Switzerland | PAVI-2017310259 | 33 | 2.550 | JBAKTX01 |
| Switzerland | PAVI-2017310266 | 22 | 2.485 | JBAKTV01 |
| Switzerland | PAVI-2017831119 | 39 | 2.551 | JBEDRY01 |
| Switzerland | PAVI-2017841322 | 29 | 2.510 | JBAKTU01 |

**Supplementary Table 3. See separate Excel File**

**Supplementary Table 4. See separate Excel File**

**Figures**

**Supplementary Figure 1. KEGG annotation of Clade 1 (A) and Clade 2 (B)-specific genes of *C. avidum***. A: 209 genes were predicted as Clade 1 specific, only 38 could be mapped to KEGG functions. B: 272 genes were predicted as Clade 2 specific, only 79 could be mapped to KEGG functions. Functional categories are shown. The analysis was done with BlastKOALA.

**A**


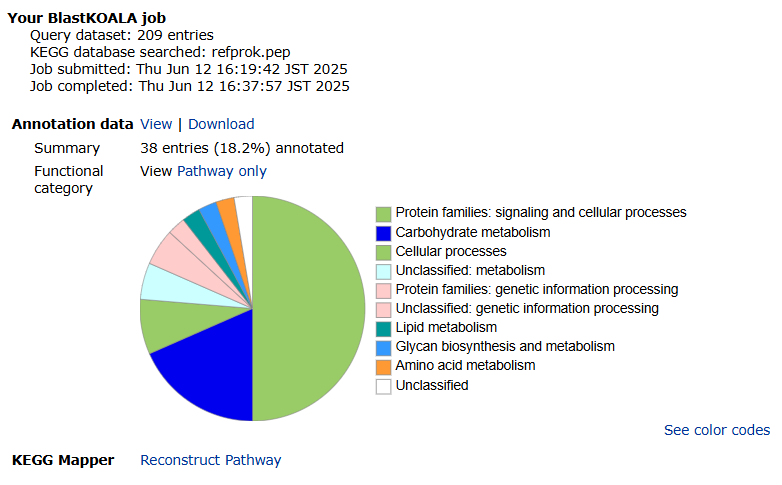


**B**


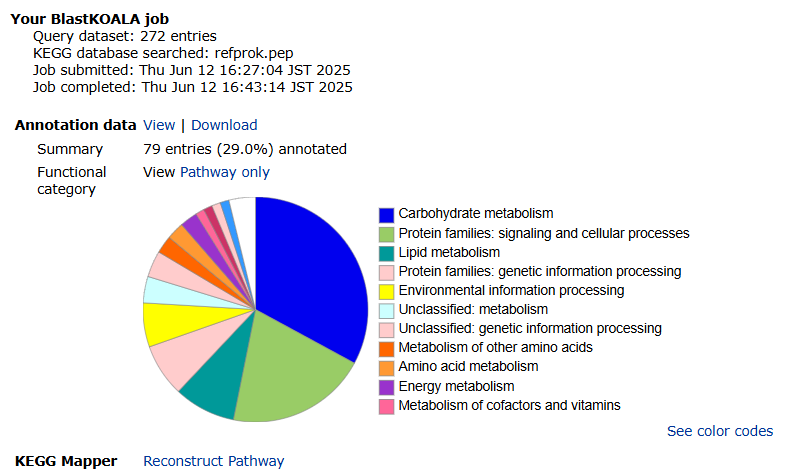

Supplement: Supplemental material — Tables S1 and S2; Fig. S1. [file spectrum.00515-25-s0001.docx]
